# Web alert: Microbial waste valorization: An annotated selection of World Wide Web sites relevant to the topics in microbial biotechnology

**DOI:** 10.1111/1751-7915.14223

**Published:** 2023-01-23

**Authors:** Lawrence P. Wackett

**Affiliations:** ^1^ Department of Biochemistry, Molecular Biology & Biophysics BioTechnology Institute, University of Minnesota St. Paul Minnesota USA

## Valorization of underutilized waste streams

### https://academic.oup.com/jimb/article/49/2/kuab056/6371101

This review covers carbohydrate‐rich and lipid‐rich wastes with a focus on nonconventional sources of waste.

## Current trends

### https://ami‐journals.onlinelibrary.wiley.com/doi/full/10.1111/1751‐7915.14198

This is an up‐to‐date review on microbial waste valorization processes.

## Valorization of plastic wastes

### https://www.frontiersin.org/articles/10.3389/fmicb.2020.00442/full

This is a general review of polymer biodegradation. While polyethylene terephthalate biodegradation is well established, there are still many questions regarding the biodegradability of polymers like high‐density polyethylene.

## Reducing biuret in commercial urea

### https://patentimages.storage.googleapis.com/a0/92/16/e9c1f7ff5f764c/US20220389465A1.pdf

Biuret is an unavoidable contaminant of urea that is used for fertilizer and diesel exhaust fluid. This patent describes a method for using microbes and enzymes to convert undesirable biuret into urea.

## Valorization of food waste

### https://www.frontiersin.org/articles/10.3389/fmicb.2020.581997/full

This review describes methods for the valorization of fruit and vegetable processing wastes.

## Valorization to produce volatile fatty acids

### https://www.mdpi.com/2311‐5637/8/9/445

This article discusses the transformation of plant and animal wastes via anaerobic fermentations into volatile fatty acids that serve as the basis for biorefineries.

## Valorization of mixed plastic waste

### https://www.nrel.gov/news/press/2022/bottle‐project‐outlines‐new‐strategy‐for‐valorization‐of‐mixed‐plastic‐waste.html

This is a commentary on research directed to the physical processing and subsequent enzymatic processing of plastic waste.

## Waste gases to products

### https://www.nature.com/articles/d41586‐022‐00488‐7

This article reports on a paper describing an ethanol producing bacterium with extra genes converting unwanted gases into products.

## Bacteria recycling electronic waste

### https://www.pnas.org/doi/10.1073/pnas.1820329116

This report describes bacteria that produce cyanide to leach metals and another bacterium that micro‐precipitates metals, principally gold.

## Trash composting

### https://www.acs.org/education/resources/highschool/chemmatters/past‐issues/2017‐2018/october2017/composting‐your‐trash‐natures‐treasure.html

This article serves as a primer for aerobic and anaerobic composting, both theoretical and practical aspects.

## Plastic waste to vanillin

### https://www.chemistryworld.com/news/genetically‐engineered‐microbes‐convert‐waste‐plastic‐into‐vanillin/4013767.article

This article describes efforts to transform polyethylene terephthalate using a recombinant *E. coli* strain to metabolize the aromatic rings forming vanillin, that is used in foods and cosmetics.

## Multi‐technology plastic valorization

### https://pubs.acs.org/doi/pdf/10.1021/acscatal.2c02775

Plastic recycling will often require a mixture of physical, chemical and biological methods to repurpose the materials. This review takes a comprehensive approach to this topic.

